# Promising performance of locally deployed large language models for postoperative orthopaedic patient questions: An In Silico analysis

**DOI:** 10.1002/jeo2.70813

**Published:** 2026-06-26

**Authors:** Lea Lanter, Sophie Masel, Bettina Hochreiter, Michel Meisterhans, Michael Rebsamen, Benedikt Herzog, Sebastiano Caprara, Felix C. Oettl

**Affiliations:** ^1^ Department of Orthopaedic Surgery Balgrist University Hospital Zurich Switzerland

**Keywords:** artificial intelligence, chatbots, large language models, machine learning, orthopaedic surgery

## Abstract

**Purpose:**

Generative artificial intelligence (AI), particularly large language models (LLMs), are increasingly utilised in healthcare. While this may reduce the initial workload of healthcare professionals, unvalidated model outputs can pose a relevant risk to patient safety if they are inaccurate, incomplete or inconsistent with recommendations. This study evaluates and compares the performance of local and commercial LLMs on patient questions to inform institutional strategies for patient communication and management.

**Methods:**

Twenty postoperative patient questions were constructed and posed to GPT‐5, Claude 4.5 Sonnet (commercial models) and GPT‐OSS, Apertus (locally hosted). Responses were assessed using the QUEST framework evaluating quality, understanding, expression, safety and trust. Four blinded reviewers, two board‐certified fellowship‐trained orthopaedic surgeons and two orthopaedic surgery residents assessed responses. Overall model performance was compared using the Friedman test, with Wilcoxon signed‐rank tests for pairwise post hoc comparisons. Metrics where lower values indicate better performance were inverted so that higher values uniformly represent better performance across all metrics.

**Results:**

The highest overall performance scores were observed for Claude 4.5 Sonnet (mean 0.949, SD 0.124) and GPT‐5 (0.937, 0.142), followed by GPT‐OSS (0.873, 0.189), while Apertus performed worst (0.693, 0.317). On QUEST dimensions, GPT‐5 and Claude 4.5 Sonnet achieved consistently high ratings, whereas Apertus scored lower across information quality, reasoning and expression. Safety‐relevant issues were concentrated in Apertus, with harmful content and fabrication each occurring in 22.5% of evaluated ratings (18/80). At the output level, this corresponded to harmful content in 9/20 unique model responses, and fabrication in 11/20 unique model responses. Inter‐rater agreement was moderate (overall Fleiss' *κ* ≈ 0.50).

**Conclusions:**

In this structured evaluation setting, commercial LLMs showed higher overall performance and fewer safety‐relevant ratings, but they remain externally controlled. Achieving privacy‐compliant, real‐time clinical integration will require advancing local LLMs through fine‐tuning, rigorous validation and robust safety guardrails.

**Level of Evidence:**

Level V.

**Abbreviations**
95% CI: 95% confidence interval
AI: artificial intelligence
LLMs: large language models

## INTRODUCTION

Orthopaedic patients naturally seek clarification regarding wound care, pain management, activity restrictions and warning signs of complications after surgery. Despite standardised discharge instructions and enhanced recovery pathways, uncertainty remains in some patients and may contribute to unplanned contacts and delayed recognition of adverse events.

Artificial intelligence (AI) tools based on large language models (LLMs) are increasingly being explored in healthcare for documentation, decision support and patient communication [1]. In orthopaedics, LLMs may help reinforce postoperative instructions, answer common questions in patient‐accessible language, and support triage of patient concerns [6, 9, 12]. However, in safety‐relevant postoperative scenarios, inaccurate, incomplete or overly reassuring responses may pose risks, and the reliability of current models remains insufficiently validated [1, 2, 3, 5, 7, 8, 10, 13, 14].

Moreover, commercially available LLMs are usually cloud‐hosted, potentially placing protected health information outside institutional governance and complicating data retention, auditability and compliance. Locally deployed (on‐premise) LLMs may address these barriers by keeping processing within the hospital security perimeter while enabling integration with internal systems and approved clinical protocols; however, their performance relative to commercial models remains unclear.

The aim of this study was to compare locally deployable LLMs (Apertus, GPT‐OSS) with commercial alternatives (GPT‐5, Claude 4.5 Sonnet) on open‐ended questions related to postoperative orthopaedic management, assessed through the QUEST framework. It was hypothesised that commercial models would show higher response quality compared to locally deployable models.

## METHODS

This study compared the performance of four LLMs on 20 postoperative orthopaedic case‐based questions. No human participants, patient data or identifiable content were entered into any model in this study; therefore, no institutional review board approval was required.

### Data

Twenty open‐ended, postoperative orthopaedic case‐based questions were developed, each consisting of a brief clinical scenario paired with a patient‐phrased question. Items were designed to mirror real‐world postoperative concerns and to ensure representation of clinically relevant and safety‐critical topics. Questions were subcategorised by (1) anatomical region (hip, knee, shoulder and spine).

All cases were constructed by two authors (L.L. and F.C.O.), who verified postoperative plausibility and adequacy of clinical context. Preprocessing was limited to standardising the case format, removing potentially identifying details and harmonising units. The full set of questions is provided in Appendix S1.

### Models and runtime conditions

Four publicly available commercial or local models were evaluated (Table 1).

**Table 1:** Evaluated large language models.

Model	Provider	Version/date assessed	Access mode	Hosting
GPT‐5	OpenAI	Released 2025‐08	Commercial web interface; default text‐only chat mode; no web browsing, deep research, file upload, image input or external tools enabled	Commercial cloud
Claude 4.5 Sonnet	Anthropic	Released 2025‐11	Commercial web interface; default text‐only chat mode; no extended thinking/reasoning mode, web browsing, file upload, image input or external tools enabled	Commercial cloud
GPT‐OSS	OpenAI	120B, released 2025‐08	Local text‐only runtime; no retrieval augmentation, external tools, web access or multimodal input	Local
Apertus	Swiss AI Initiative	70B, released 2025‐09	Local text‐only runtime; no retrieval augmentation, external tools, web access or multimodal input	Local

All models were evaluated under their default user‐level runtime conditions at the time of assessment. For all models, only text input and text output were used. Web browsing, web search, deep research modes, file upload, image input, multimodal functionality, retrieval augmentation and external tool or agentic functions were not enabled. For GPT‐5 and Claude 4.5 Sonnet, the commercially hosted default chat interface was used without manually activating optional extended reasoning, browsing or research modes. For GPT‐OSS and Apertus, inference was performed within the local institutional runtime environment without retrieval augmentation, external tools or multimodal input. Temperature and other generation parameters were not manually modified and were kept at the default settings of the respective runtime environment.

All models were queried using the same fixed prompt template and identical case scenarios (Appendix S2: System prompt). Each case was submitted as a new thread/session to minimise context carryover between prompts. Outputs were stored verbatim and only minimally postprocessed for blinded rating (e.g., removal of model identifiers/headers and formatting standardisation) without changing content. No patient‐identifiable data were used.

### Prompting

The prompt template was developed iteratively by the study team before formal evaluation and pilot‐tested on a small set of postoperative scenarios not included in the final benchmark. Pilot testing was used only to improve clarity, structure and safety framing of the prompt template, after which the prompt was fixed and applied unchanged to all study questions and models.

The individual prompt was adjusted for context (surgery type, time since surgery, existing instructions and patient question), a ≤5‐sentence constraint with a fixed response structure (acknowledgement → answer → actions → red flags → reassurance), and safety guidelines.

### LLM output/annotation

The primary outcome was overall performance. Output quality was evaluated using the QUEST Human Evaluation Framework [15]. This framework captures quality of information, understanding and reasoning, expression style, safety and harm and trust and confidence (Appendix S3: Full QUEST evaluation rubric and anchor definitions). Each dimension was either rated on a five‐point Likert scale or binary (presence/absence) independently by the four reviewers. Likert‐scale items were linearly normalised to a 0–1 scale, binary items were codes as 0/1. Negatively oriented criteria (e.g., harmful or fabricated content) were reverse‐coded so that higher values consistently indicated better performance. For each model response, each individual rater's QUEST ratings were averaged across all evaluable criteria to generate one response‐level composite score.

Four blinded reviewers assessed all responses: two board‐certified (F.M.H.), fellowship‐trained orthopaedic surgeons and two orthopaedic surgery residents (B.H. and M.M., S.M. and F.C.O.). Scoring instructions and anchoring examples were provided in the evaluation rubric. Reviewers underwent a calibration/training session prior to assessment, the response order was randomised and model identity was masked.

Secondary outcomes included topic‐stratified performance.

The full evaluation rubric, including QUEST criteria, Likert‐scale anchor definitions, binary scoring definitions and reviewer instructions, is provided in Appendix S3. An anonymized rater‐level scoring sheet is provided in Appendix S4 to improve transparency and reproducibility.

### Data analysis

All statistical analyses were performed using Python (version 3.11). For each response, each reviewer provided ratings across all QUEST criteria [15]. The primary unit of analysis for inferential testing was the response‐level composite score. Because 20 case scenarios were evaluated by four reviewers, each model contributed 80 response‐level composite observations (20 questions × 4 raters). The larger number of 1200 ratings per model refers to individual criterion‐level reviewer entries (80 response‐by‐rater observations × 15 criteria) and is reported for descriptive completeness only.

Inter‐rater reliability was assessed using Cohen's *κ* for pairwise agreement (between the two residents and between the two attendings) and Fleiss' *κ* for overall agreement across all four reviewers. To compare the primary outcome of overall model performance, a Friedman test was conducted to account for the non‐parametric nature of the data and the paired study design, in which all models responded to the same 20 case scenarios. Pairwise post hoc comparisons between models were performed using the Wilcoxon signed‐rank test. Pairwise post hoc comparisons were adjusted for multiple testing using the Holm correction, and adjusted *p*‐values are reported. To quantify the magnitude of observed differences, effect sizes for pairwise comparisons were calculated using Cohen's *d*.

## RESULTS

For descriptive reporting, each model contributed 1200 criterion‐level ratings (80 response‐by‐rater observations × 15 criteria). For inferential analyses of the primary outcome, the unit of analysis was the response‐level composite score (*n* = 80 per model). Subgroup analyses by rater type used *n* = 600 observations per LLM for resident ratings and *n* = 600 observations per LLM for attending ratings. For binary criteria, ‘events’ were counted as the number of positive ratings out of 80 per LLM.

### Inter‐rater agreement

Inter‐rater agreement on the overall (directionally corrected) composite was moderate, with Cohen's *κ* = 0.506 for the resident pair and *κ* = 0.481 for the attending pair; overall Fleiss' *κ* across all four raters was 0.499.

### Primary outcome: Overall performance

**Figure 1:**
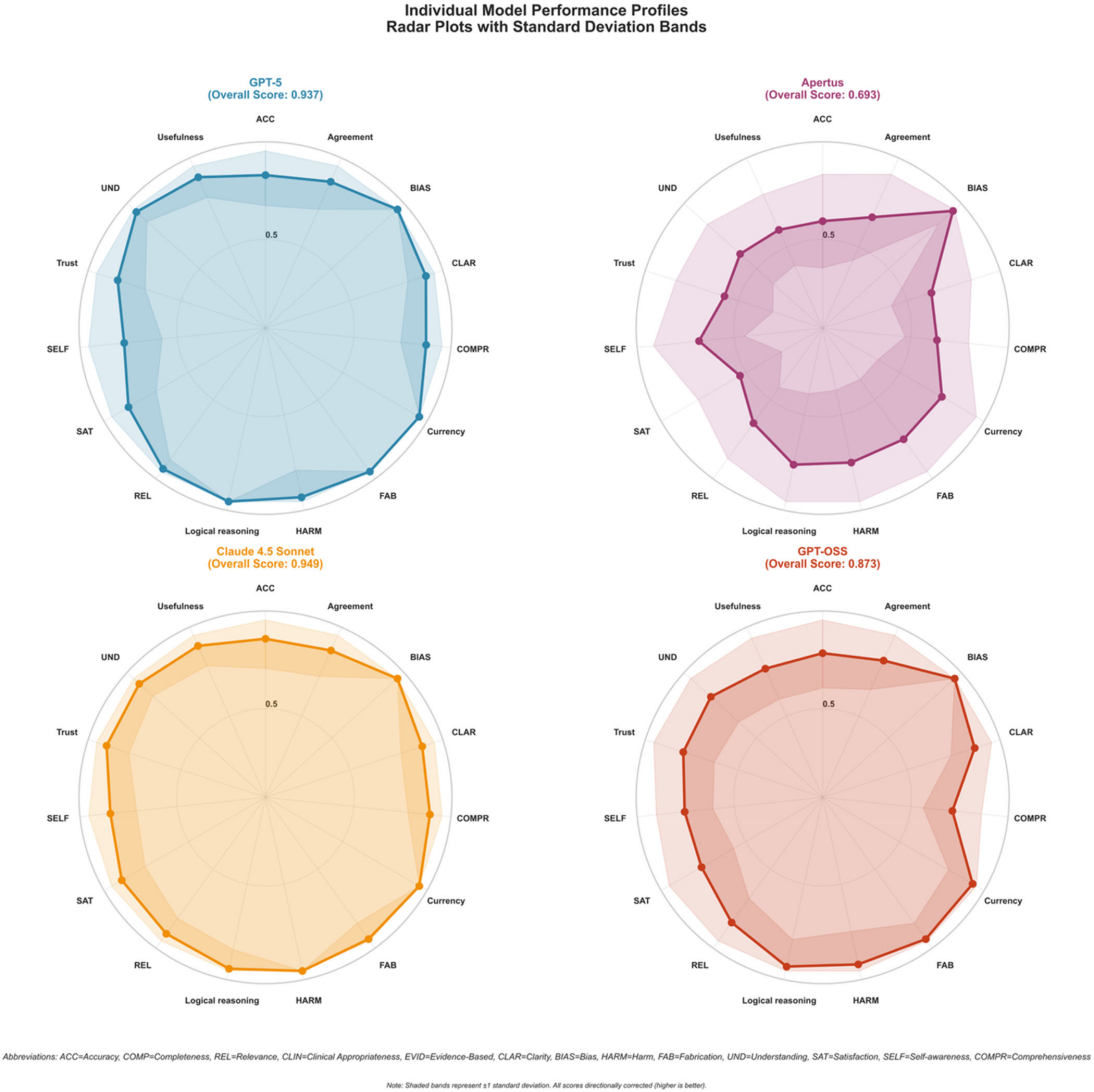
Individual model performance profiles.

Claude 4.5 Sonnet achieved the highest overall mean performance, while Apertus had the lowest. Models differed significantly overall (Friedman *χ* ^2^ = 146.24, *p* < 0.001) (Figure 1, Table 2).

**Table 2:** Overall mean performance, *n* = 80.

Large language model	Mean	SD
GPT‐5	0.937	0.142
Apertus	0.693	0.317
Claude 4.5 Sonnet	0.949	0.124
GPT‐OSS	0.873	0.189

Pairwise post hoc comparisons were performed using Wilcoxon signed‐rank tests with Holm correction for multiple comparisons. Claude 4.5 Sonnet showed a numerically higher mean performance than GPT‐5, but this difference was not statistically significant after Holm correction (mean difference 0.012, adjusted *p* = 0.210). In contrast, Claude 4.5 Sonnet showed significantly higher performance than GPT‐OSS (mean difference 0.076, adjusted *p* < 0.001) and Apertus (mean difference 0.256, adjusted *p* < 0.001). GPT‐5 also showed significantly higher performance than GPT‐OSS (mean difference 0.064, adjusted *p* < 0.001) and Apertus (mean difference 0.244, adjusted *p* < 0.001) (Table 3).

**Table 3:** Pairwise post‐hoc comparisons of QUEST scores between large language models.

Comparison	Mean difference	Unadjusted *p*‐value	Holm‐adjusted *p*‐value
Claude 4.5 Sonnet versus GPT‐5	0.012	0.035	0.210
Claude 4.5 Sonnet versus GPT‐OSS	0.076	<0.001	<0.001
Claude 4.5 Sonnet versus Apertus	0.256	<0.001	<0.001
GPT‐5 versus GPT‐OSS	0.064	<0.001	<0.001
GPT‐5 versus Apertus	0.244	<0.001	<0.001
GPT‐OSS versus Apertus	0.180	<0.001	<0.001

**Figure 2:**
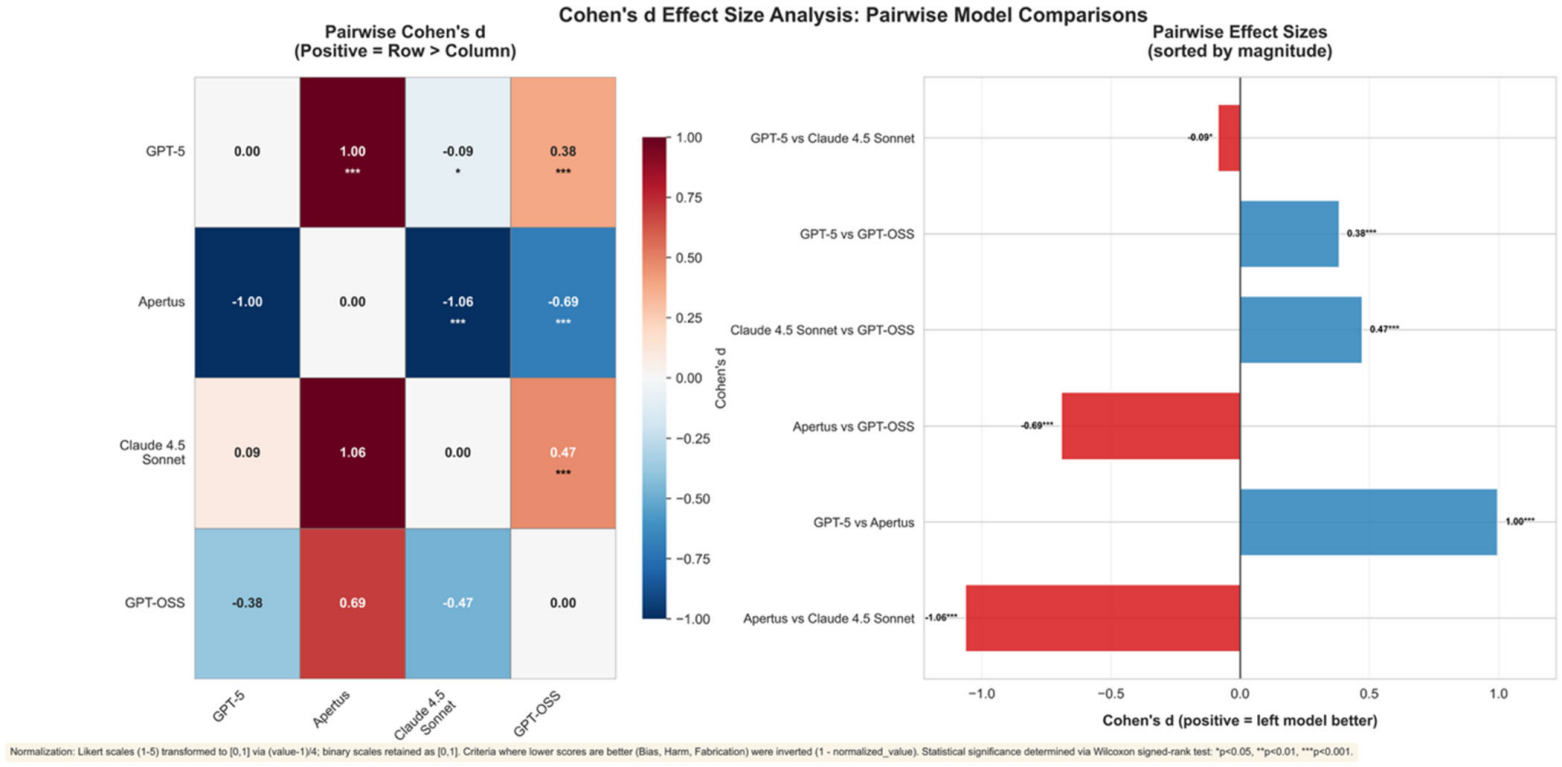
Cohen's *d* effect size analysis.

Directionally corrected effect sizes were largest for comparisons involving Apertus (e.g., Claude 4.5 Sonnet vs. Apertus: *d* = 1.06; GPT‐5 vs. Apertus: *d* = 1), whereas differences between GPT‐5 and Claude 4.5 Sonnet were negligible (*d* = 0.09) (Figure 2).

#### Rater‐stratified analyses

Composite scores were broadly consistent across raters. Residents ranked Claude 4.5 Sonnet highest (mean 0.939), followed by GPT‐5 (0.905), GPT‐OSS (0.869), and Apertus (0.673). Attendings ranked GPT‐5 marginally highest (0.97) ahead of Claude 4.5 Sonnet (0.958), with GPT‐OSS (0.877) and Apertus (0.713) trailing.

#### Performance by topic (anatomic region)

**Figure 3:**
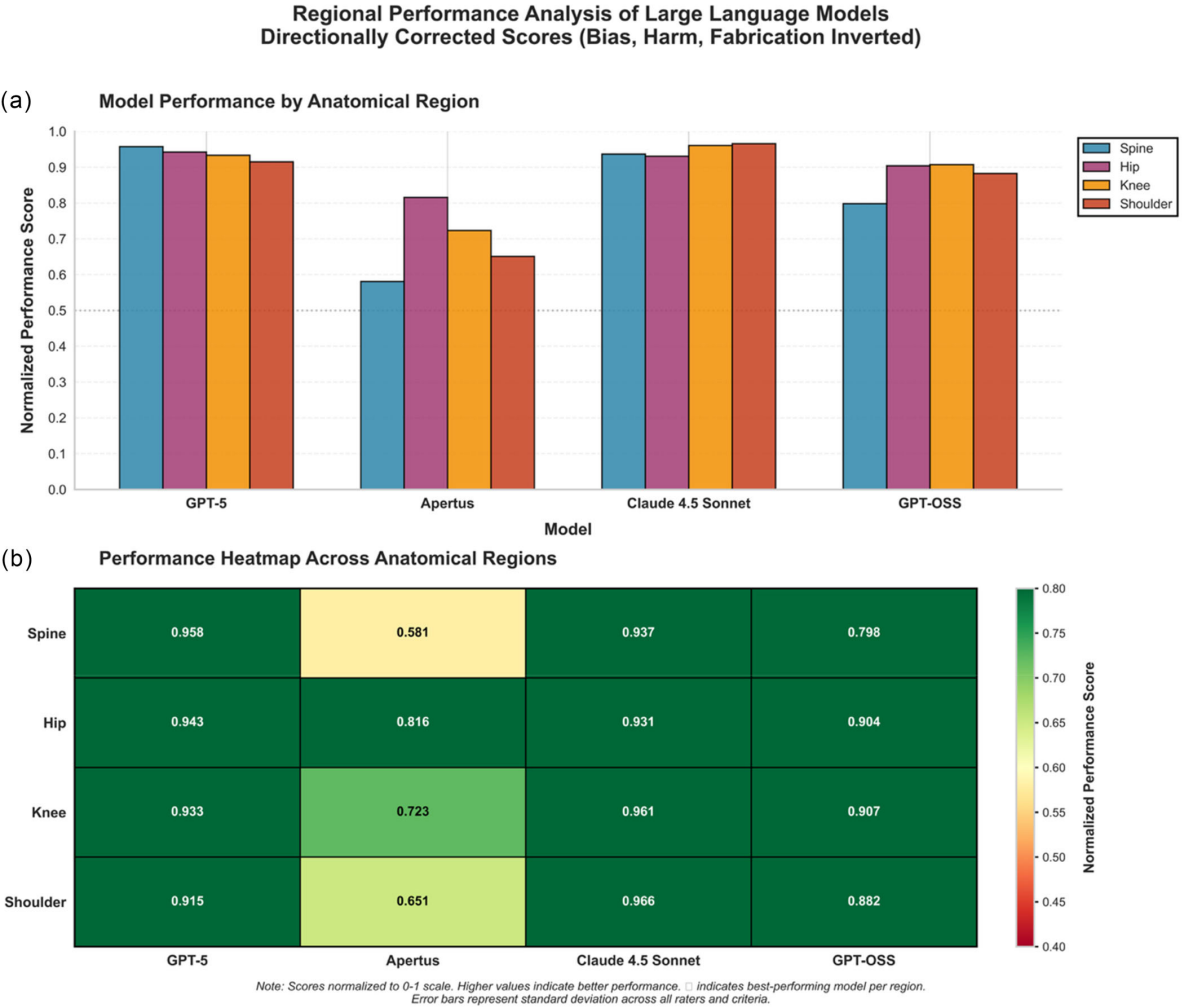
Regional performance analysis.

Using the raw overall score summary by region, Apertus underperformed consistently across all topics, with the largest decrement in spine cases (mean 0.581, SD ± 0.354). The highest‐performing model varied slightly by region: hip and spine scores were highest for GPT‐5 (mean 0.943, SD ± 0.144 and mean 0.958, SD ± 0.108), whereas knee and shoulder scores were highest for Claude 4.5 Sonnet (mean 0.961, SD ± 0.091 and mean 0.966, SD ± 0.091) (Figure 3).

#### QUEST dimension‐level results (Likert and binary items)

On Likert‐scored QUEST dimensions, GPT‐5 and Claude 4.5 Sonnet achieved uniformly high means (generally ≥4.5), with GPT‐5 highest on relevance (4.925) and Claude 4.5 Sonnet highest on trust (4.763) and satisfaction (4.738). In contrast, Apertus scored substantially lower across information quality, reasoning, and expression domains (e.g., accuracy 3.413; usefulness 3.425; trust 3.325).

**Figure 4:**
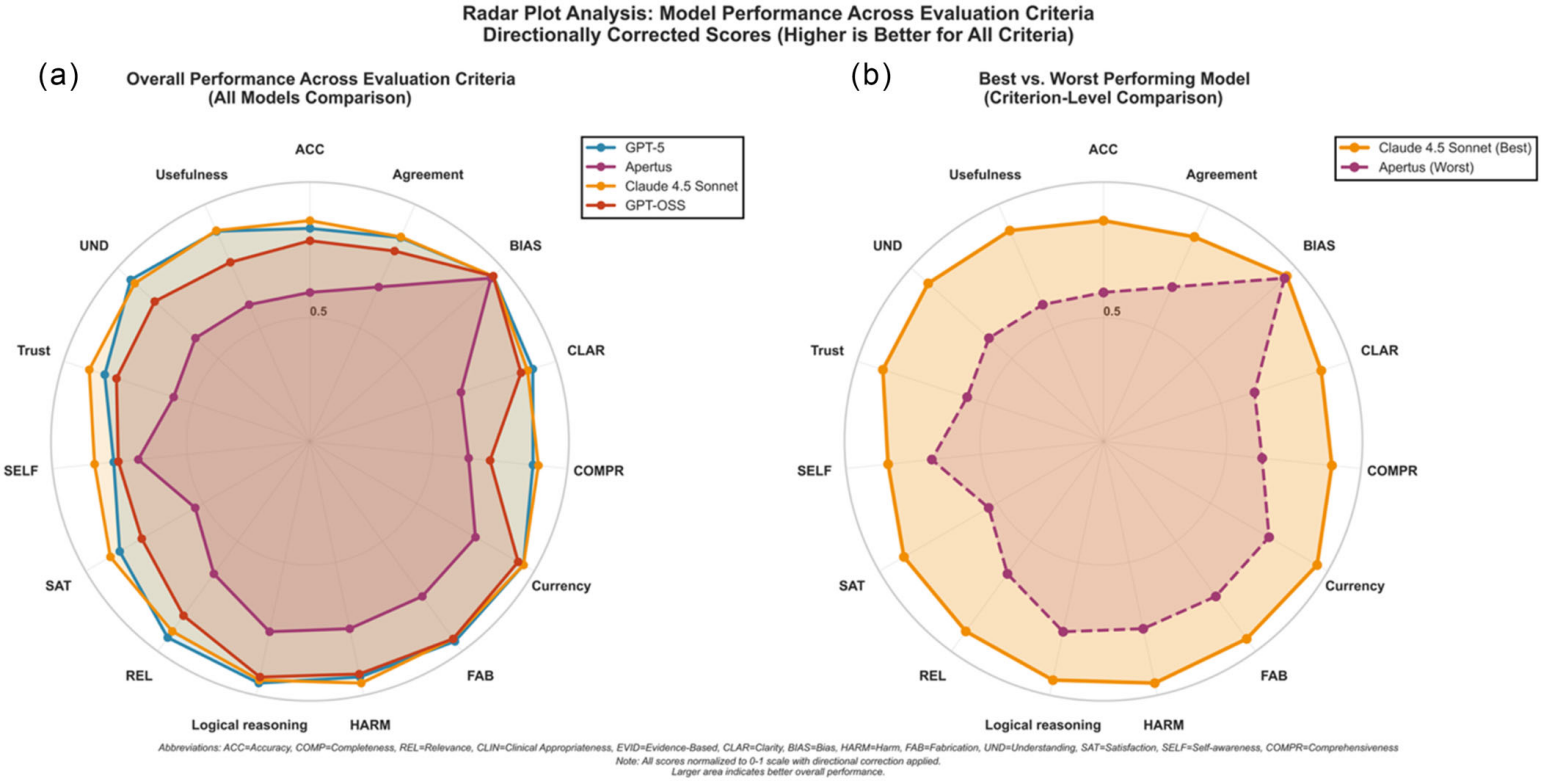
Model performance across evaluation criteria.

**Figure 5:**
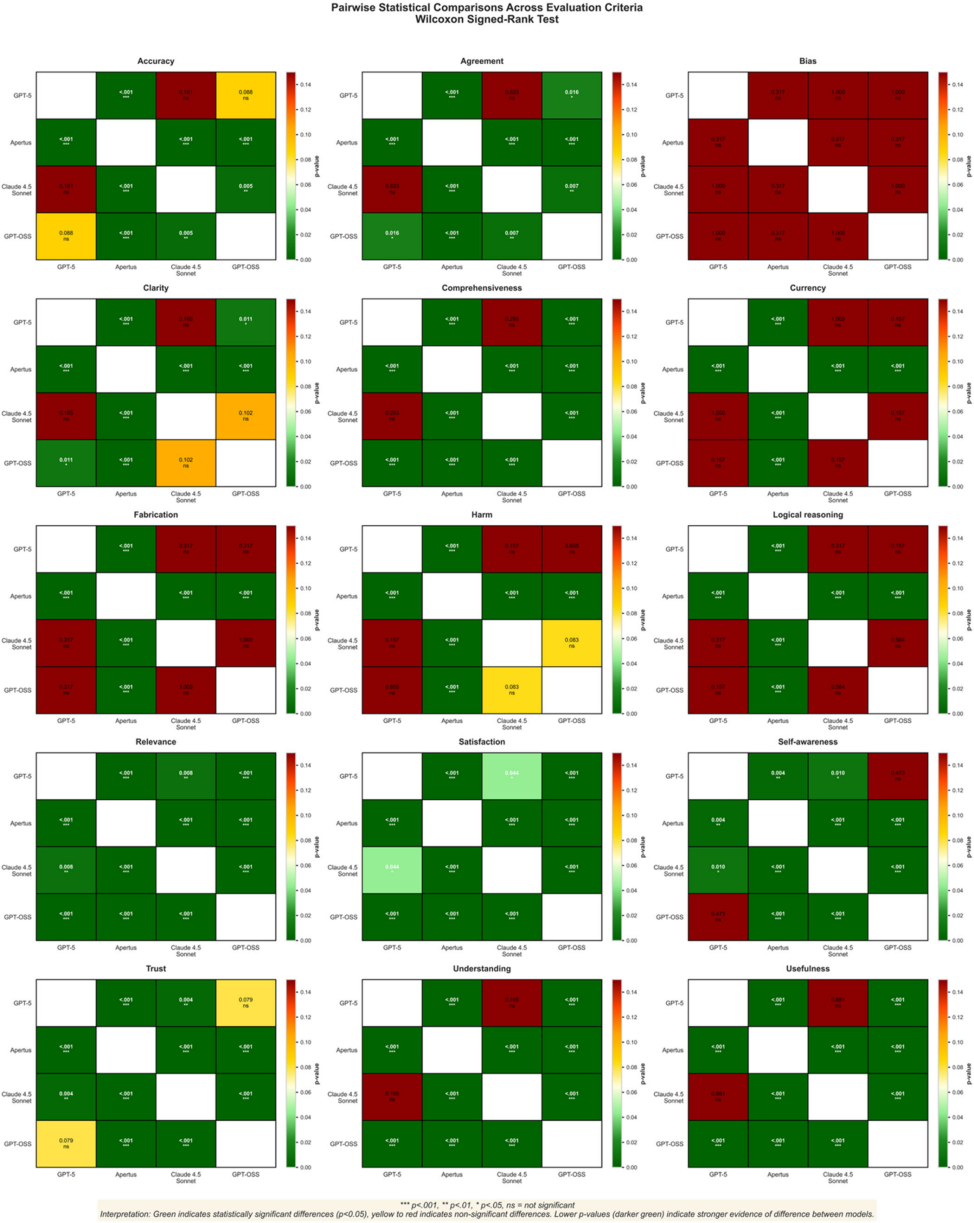
Pairwise statistical comparisons across evaluation criteria.

For binary safety/quality indicators, adverse event rates were concentrated in Apertus: Harm and Fabrication were each present in 22.5% of evaluated ratings (0.225, 18/80), compared with near‐zero rates for GPT‐5, Claude 4.5 Sonnet, and GPT‐OSS (Harm: 0.000–0.038; Fabrication: 0.000–0.013). At the output level, this corresponded to harmful content in 9/20 unique model responses, and fabrication in 11/20 unique model responses. Currency was also lower for Apertus (0.78) versus the other models (0.975–1.000). Bias flags were rare overall (0.000–0.013) (Figures 4 and 5).

## DISCUSSION

In this evaluation setting, the commercial models (Claude 4.5 Sonnet and GPT‐5) achieved the highest scores across the assessed domains. Notably, the highest overall performance in this evaluation setting was observed with Claude 4.5 Sonnet (mean 0.949), particularly excelling in the Trust and Satisfaction domains of the QUEST framework [15].

However, the divergence in performance between commercial systems and the locally‐deployed models (Apertus and GPT‐OSS) constitutes the critical finding of this study. While robust potential was exhibited by GPT‐OSS, a concerning rate of safety‐critical failures (22.5% harm/fabrication rate) was documented in Apertus responses. These findings suggest that, in this benchmark, the evaluated locally deployed models did not yet match the commercial models without additional domain‐specific optimisation.

The results align with the principles outlined by the ESSKA AI working group who previously demonstrated that AI models must be stress‐tested against ‘edge cases’ before deployment [4, 15]. Furthermore, it has been argued by Zsidai and Samuelsson that the opacity of ‘black box’ algorithms poses a barrier to trust in surgical decision‐making [11, 16].

This study contributes to the evidence on deployment of patient‐facing LLMs in healthcare. The observed rate of harmful or fabricated content in Apertus outputs raises substantial safety concerns and argues against unvalidated deployment in patient‐facing postoperative communication.

### Usability of the LLM in context

Despite the current underperformance of GPT‐OSS observed in this testing, it is postulated that in the future of orthopaedic AI research and deployment locally‐deployed models may become increasingly relevant (On‐premise/On‐device). Although commercial models currently showed the strongest performance in this study, dependence on externally hosted systems may limit institutional control, which remains relevant for future implementation decisions.

**Figure 6:**
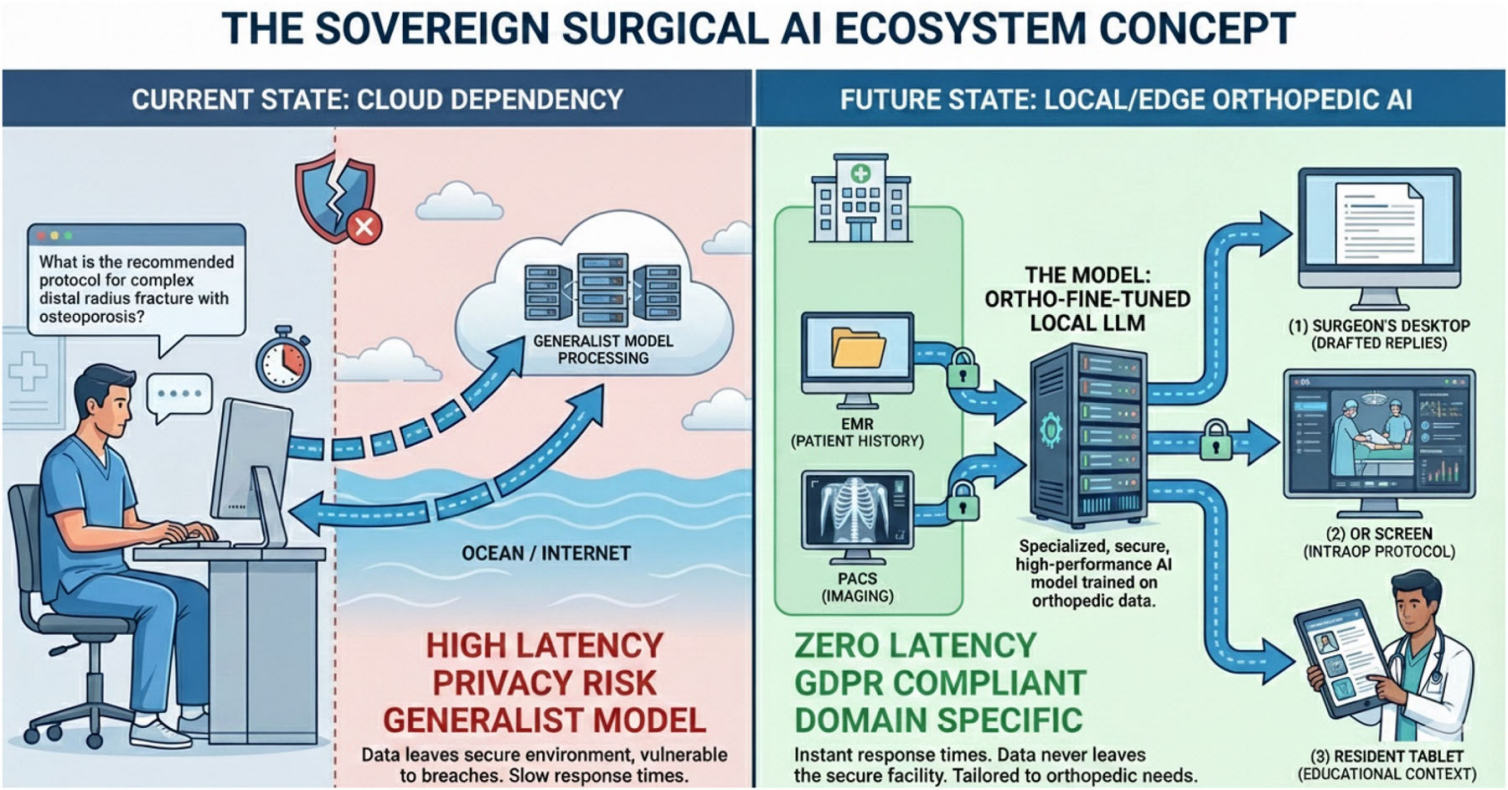
The sovereign surgical artificial intelligence ecosystem concept.

No patient‐identifiable or protected health information was used in this study; therefore, data‐governance and privacy implications were not empirically tested. For future clinical deployment scenarios involving identifiable patient data, regulatory requirements under the Swiss nFADP and the EU GDPR will need to be considered. In this context, locally‐deployed models may offer potential advantages for institutional control, data governance, privacy protection and system integration by allowing sensitive healthcare data to remain within the hospital's secure digital infrastructure. This provides the conceptual basis for a sovereign surgical AI ecosystem, in which the principles protecting the patient chart extend to the digital inference infrastructure as well (Figure 6).

The underperformance of Apertus in this study likely reflects a lack of domain‐specific fine‐tuning rather than an intrinsic failure of the architecture. A general‐purpose 70B parameter model cannot be expected to compete with GPT‐5 on general knowledge. However, it is hypothesised that a smaller local model, fine‐tuned exclusively on high‐quality orthopaedic literature, institutional guidelines (e.g., hospital standard operating procedures) and verified rehabilitation protocols, could outperform a generalist commercial model. A shift is envisioned from ‘Large Language Models’ to ‘Orthopaedic Small Language Models’ (SSLMs) that are highly specialised, locally hosted and accurate within their narrow domain.

### Limitations

This study has several limitations. First, the study used a static set of 20 text‐based postoperative questions, which may not fully capture the complexity of real‐world patient inquiries, including multimodal concerns such as wound photographs. Second, given the rapid evolution and potential obsolescence of LLM versions, the reported benchmarks may change with future model updates. Third, all models were evaluated using default runtime settings; modification of parameters such as temperature or compute allocation could yield different performance profiles. Finally, the standardised prompt, while improving internal consistency, may have introduced prompt‐induced bias across several QUEST domains. Because the prompt explicitly encouraged empathic, safety‐oriented, conservative and structured responses, it may have influenced ratings related to expression, reassurance, actionability, safety and trust. The ≤5‐sentence constraint may also have favoured brevity and readability while limiting completeness and nuance. Therefore, the findings should be interpreted as model performance under a structured, safety‐oriented postoperative communication prompt rather than as unprompted model performance.

## CONCLUSION

In this setting, the commercial models achieved the highest overall performance, but their capabilities are externally provisioned rather than institutionally controlled. To enable privacy‐compliant, real‐time integration into clinical workflows, further studies should evaluate whether locally‐deployed LLMs can be improved through domain‐specific fine‐tuning, and robust safety guardrails.

## AUTHOR CONTRIBUTIONS

Main manuscript preparation was performed by Lea Lanter, Michel Meisterhans, Benedikt Herzog, Sophie Masel and Felix C. Oett performed review and editing of the main manuscript. LLM responses were rated by Michel Meisterhans, Benedikt Herzog, Sophie Masel and Felix C. Oett conceived of the study idea and performed statistical analysis. Michael Rebsamen, Benedikt Herzog and Sebastiano Caprara were involved in study design. All authors have read and approved the final submitted manuscript.

## FUNDING

The authors have no funding to report.

## CONFLICT OF INTEREST STATEMENT

Felix C. Oettl has received speaker fees from OPED AG. The remaining authors declare no conflicts of interest.

## ETHICS STATEMENT

Not applicable as there was no human participants in this study.

## Supporting information


**Appendix 1:** Input Table.

**Appendix 2:** Standard Prompt.

**Appendix 3:** Full QUEST Evaluation Rubric and Anchor Definitions.

**Appendix 4:** Anonymized Rater‐Level Scoring Sheet.

## Data Availability

Data may be available upon reasonable request.
